# 

**Published:** 1897-05-08

**Authors:** 


### EARLY EDITION.

### NOTICE TO CORRESPONDENTS.

All MS., Letters, Books for Review, and other matters intended for the Editor should be addressed The Editor, "The Hospital," The Hospital Building, 28 & 29, Southampton Street, Strand, W.C.

The Editor cannot undertake to return rejected MS., even when accompanied by stamped directed envelope.

**Notice to Advertisers and Subscribers.**

All Advertisements, Orders for Copies of The Hospital, and Business Communications should be addressed to **THE MANAGER, (Not to The Editor),** The Hospital, The Hospital Building, 28 & 29, Southampton Street, Strand, London, W.C.

The Cost of Subscription, post free, is as follows:—Per week, 2½d.; per quarter, 2s. 8d.; per half-year, 5s. 3d.; per annum, 10s. 6d. Foreign:—Per annum, 15s. 2d.; payable, in all cases, in advance.

**NOTICE.—Vol. XXI. of "The Hospital" (October, 1896, to March, 1897), in handsome cloth binding, is on sale at the Office of this Journal, price 7s. 6d. Cases for binding the Numbers contained in this Volume, 1s. 6d. Index to Vol. XXI., 2½d., post free.**

**The Monthly Part for May is now ready. Price 6d. by post 8d.**

### The Student of Medicine.

### APPOINTMENTS.

S. B. Blomfield, L.S.A., appointed Medical Officer for the Hurst Green District of the Ticehurst Union. J. H. Crocker, M.D., D.P.H., appointed Medical Officer of Health of the Manchester Port Sanitary Authority. J. Edgar P. Davies, M.B., B.Sc.Lond., M.R.C.S., L.R.C.P.Lond., appointed Medical Officer to the South Wales Steel and Tinplate Works, Llanelly. A. Francis Dixon, B.A., M.B.Dub., appointed Professor of Anatomy at the University College, Cardiff. A. E. Griffin, M.A.Cantab., M.R.C.S.Eng., L.R.C.P.Lond., appointed House Physician to the Dreadnought Seaman's Hospital, Greenwich, S.E. W. A. Hayes, M.R.C.S.Eng., L.R.C.P.Lond., appointed Medical Officer to the Cape Government Railways at Mafeking, British Bechuanaland. Albert Henderson, M.A., M.B.Aberd., appointed Medical Officer to the Aberdeen Dispensary. Mr. Kennedy, appointed Assistant Medical Officer to the Enniscorthy Lunatic Asylum. L. F. B. Knuthsen, appointed Surgeon-on-Duty at the Falmouth Hospital. Fred. Porter, M.B., C.M.Edin., appointed Assistant Medical Officer to Roxburgh District Asylum, Melrose. A. J. Rodocanachi, M.D.Lond., appointed House Surgeon to the District Infirmary, Ashton-under-Lyne. Lionel Smith, M.R.C.S., L.R.C.P., appointed Resident Medical Officer, Westminster General Dispensary. W. D. Watson, M.R.C.S., L.R.C.P., appointed Medical Officer for the Leigh District of the Rochford Union. Douglas Seaton, M.B., Ch.B., appointed Resident Surgical Officer, Leeds General Infirmary. E. J. Lumb, M.R.C.S., L.R.C.P., appointed one of the House Physicians, Leeds General Infirmary. E. J. A. Dodd, M.R.C.S., L.R.C.P., appointed one of the House Surgeons, Leeds General Infirmary. F. E. Taylor, M.A., B.Ch., M.B., appointed one of the House Surgeons, Leeds General Infirmary. J. V. Hartley, M.R.C.S., L.R.C.P., appointed Resident Medical Officer at Ida Hospital, Cookridge, near Leeds. John Fawcett, M.D.Lond., M.R.C.P., F.R.C.S., appointed Assistant Physician, Royal Free Hospital. R. H. P. Crawford, M.A., M.D., M.R.C.P., appointed Pathologist, Royal Free Hospital. R. P. Williams, M.B., appointed House Physician, Royal Free Hospital.

